# The Thermoregulatory Behavior of Nectar Foraging Polistine Wasps (*Polistes dominula* and *Polistes gallicus*) in Different Climate Conditions

**DOI:** 10.3390/insects10070187

**Published:** 2019-06-27

**Authors:** Helmut Kovac, Helmut Käfer, Anton Stabentheiner

**Affiliations:** Institute of Biology, University of Graz, 8010 Graz, Austria

**Keywords:** Polistes, thermoregulation, foraging, operative temperature, climate

## Abstract

Polistine wasps collect nectar for their energetic demand and for the provision of the brood. They are mainly ectothermic during different behavioral tasks. We investigated the body temperature of two species living in differing habitats and climate regions, in order to reveal the environmental influence on their thermoregulatory behavior. The species were *Polistes dominula* in the temperate climate of Central Europe, and *Polistes gallicus* in the warm Mediterranean climate of Southern Europe. The wasp’s body temperature was measured during foraging on lovage (*Levisticum officinale*) and fennel (*Foeniculum vulgare*) by infrared thermography in the entire ambient temperature range they are usually exposed to (T_a_ ~ 20–40 °C). The temperature of all body parts increased nearly linearly with ambient temperature, with the thorax as the warmest part. To achieve optimal foraging temperatures, they preferably use solar radiation. An “operative temperature model” enabled the evaluation of the endothermic effort. *Polistes dominula* foraging on lovage exhibited no endothermic activity. However, while foraging on fennel they had a weak and almost constant endothermic performance of about 1 °C. *Polistes gallicus*, by contrast, exhibited mostly no or only minor endothermy during foraging. Both wasps avoid a high energetic effort and this way reduce their foraging costs.

## 1. Introduction

Polistine wasps exhibit a “low-level energetic” lifestyle. They spend a lot of time nearly immobile at their nests, and they are mainly ectothermic during different behavioral tasks at the nest. They prey on other insects and collect nectar for their own energetic demand and for provision of the brood. During foraging, they are exposed to a broad range of ambient temperatures and solar radiation conditions. These environmental conditions have a great influence on their body temperature [1]. In contrast to Vespine wasps, which achieve a high agility by means of endothermic heat production [1,2], the body temperature of Polistine wasps depends much more on the ambient temperature. In certain cases, however, it was found to be elevated above the ambient temperature to some extent, for example, during flight at laboratory conditions [3,4], during alert or nest defense [5], and when collecting water [1]. While it is clear that an insect of the size of a Polistes wasp is endothermic during flight, e.g., [6,7,8,9], because of the high flight energy expenditure [7], it is unknown whether they improve their agility during foraging for nectar by an elevated thoracic temperature. The question arises, whether an elevation of the body temperature is achieved by endothermic heat production of the wasps, or by heat gain due to solar radiation, or both. In insects with little or no endothermic activity, the use of solar radiation is highly important for thermoregulation [6].

*Polistes dominula* and *Polistes gallicus* are closely related species of paper wasps with a similar body shape, but differing size. Both collect nectar on similar plants, preferably on flowers of Apiales. *Polistes dominula* has a wide distribution range covering different climate regions, whereas *Polistes gallicus* is restricted to the Mediterranean area in its distribution. In general, in evolutionary adaptation processes species have developed special physiological features to cope with the environmental conditions of their habitat. In *Polistes dominula* and *Polistes gallicus,* it could be shown that these species have developed thermal traits according their thermal environment [10]. The aim of this study was to investigate the thermoregulatory behavior of these two closely related species in different habitats with differing climate, under special consideration of the microclimate conditions. The wasps’ body temperature was measured during foraging for nectar on lovage and fennel in their typical habitat in the temperate climate of Central Europe (Austria) and in the Mediterranean climate of Tuscany (Italy). Our study should reveal the environmental influence on thermoregulatory aspects during foraging and should reveal the most important parameters influencing the wasps’ body temperature. The energetic demand in ectotherms increases with ambient temperature, and a rising temperature due to global warming will be a challenge for their energy balance. For modelling the energetic effort of ectotherms (under future climate conditions), it is of great advantage to know the insect’s body temperature. Our investigation delivers the necessary data. In the context of climate change, such investigations become more and more important.

## 2. Materials and Methods

### 2.1. Animals and Study Sites

We investigated the common and widespread paper wasp *Polistes dominula*, with a mean body size of 80 ± 15 mg, and the closely related *Polistes gallicus* of a somewhat smaller size of 44 ± 11 mg [10]. Although the two species differ in their size, they are quite similar in body shape and their yellow and black coloration. The measurements on *Polistes dominula* were conducted in Gschwendt/Kumberg, Austria (47°10′41.59″ N, 15°34′22.59″ E), in the temperate climate region of Central Europe. The thermal behavior of *Polistes gallicus* was investigated in Sesto Fiorentino/Florence, Italy (43°49′2.59″ N, 11°12′4.48″ E) in the Mediterranean climate region of Southern Europe. To describe the climatic variability of the two regions of the study sites, data of the meteorological stations for the standard reference period from 1971–2000 of Graz (N 47°04′40″, E 15°26′56″) and Florence (N 43°46′28″, E 11°15′18″) were evaluated. Origin of data for Graz: Zentralanstalt für Meteorologie und Geodynamik (http://www.zamg.ac.at/fix/klima/oe71-00/klima2000/klimadaten_oesterreich_1971_frame1.htm), and for Florence: Consorzio LaMMA (http://www.lamma.rete.toscana.it/clima-e-energia/climatologia/clima-firenze-1971-2000). The mean annual temperature for the thirty-year period was 9.4 °C in Graz and 14.9 °C in Florence. The locations differed also in the mean maximum and minimum temperatures (Graz: mt_max_ = 14.6 and mt_min_ = 5.5 °C, Florence: mt_max_ = 20.5 and mt_min_ = 9.3 °C; [10]).

The investigations were conducted on two different herbs, lovage (*Levisticum officinale*) and fennel (*Foeniculum vulgare*). These are two similar Apiales with the typical habitus of the Apiales with branched inflorescences and small yellow flowers and a height of about 1.5 m. We chose these flowers as they are common and widespread, and the wasps are frequently observed to forage at these plants, and we presumed that they are very attractive for them. *Polistes dominula* was measured on three days in June in 2017 in a garden, where they were foraging for nectar on lovage and on two days during foraging on fennel in August. In Italy we investigated *Polistes gallicus* on two days in July in 2017, in a park during foraging for nectar on fennel. We started the measurements early in the morning when the first wasps appeared at the flowers and measured until the evening.

### 2.2. Body Temperature Measurement and Environmental Conditions

The surface temperature of the wasps’ body (head, thorax, and abdomen) was measured by infrared thermography (T650sc, FLIR Systems Inc., Danderyd, Sweden) during a foraging bout. The measured body temperature was calibrated to 0.7 °C accuracy, assuming a wasp cuticle infrared emissivity of 0.97 [11] and using a proprietary Peltier driven reference source of known temperature and emissivity for the camera calibration [11,12,13,14]. The infrared data were stored digitally on an internal memory card at a frame rate of 30 Hz and evaluated afterwards in the laboratory. The evaluation of the surface temperatures of the head (T_head_), thorax (T_thorax_), and abdomen (T_abdomen_) was done with the AGEMA Research software (FLIR Systems Inc., Wilsonville, OR, USA) controlled by a proprietary Excel VBA macro (Microsoft Corporation, Redmond, WA, USA).

The environmental conditions, micro- and macroclimate in the habitat were recorded during the entire measurement period. *Microclimate:* The following data were logged at 1 s intervals with data loggers (ALMEMO 2690, Ahlborn GmbH, Holzkirchen, Germany): the ambient temperature (T_a_) beside the wasps (within ~1–2 cm) and in the inflorescence (T_flower_) with thermocouples (K-type; the readout corrected for the effect of solar radiation), the humidity (with FHA646-R, Ahlborn, Holzkirchen, Germany), and the solar radiation (with a global radiation sensor FLA613GS/Mini spezial, measurement range of 380–1100 nm; Ahlborn, Holzkirchen, Germany) in the inflorescence. *Macroclimate:* Two meters beside the measuring sites we installed a simple weather monitoring station to measure ambient temperature (T_globe_), relative humidity, solar radiation, and wind speed in the habitat under standardized conditions.

### 2.3. Operative Temperature Model

**Figure 1:**
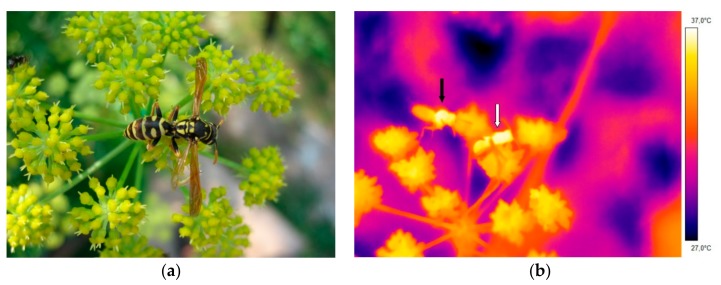
(**a**) Dead wasp (*Polistes dominula*) attached to an inflorescence of lovage for measuring the operative temperature (T_e_). (**b**) Thermogram of a living wasp (left, black arrow) foraging on lovage and a dead wasp (right, white arrow), measured at an ambient temperature of 30 °C.

To take into consideration the effects of ambient air temperature, solar radiation, and air convection of the measurement site on the wasps’ body temperature, we determined the insects’ thermal environment, expressed in terms of the operative (environmental) temperature (T_e_). The operative temperature is the temperature of an object with the same external (e.g., size, shape, and color) and internal characteristics as the animal under consideration; it integrates convective and radiative heat transfer between the environment and the animal [1,15,16,17,18]. The use of fresh insect carcasses is highly recommended in such studies, because they represent the passive reaction to environmental conditions much better than dried specimens [1,19]. In our study, a dead wasp was used as an operative temperature thermometer. For this purpose, a wasp was caught and killed by freezing. The defrosted wasp was attached to the center of the flowers’ inflorescence in a natural foraging position, as shown in Figure 1a. The body surface temperature of the dead wasp was measured by infrared thermography (see above) and the ambient temperature (T_a_) was measured 1 cm beside the wasp, as shown in Figure 1b. The dead wasp was measured simultaneously or in alternation with the living foraging wasps. With this model, we were able to quantify the part of temperature increase generated by endothermic heat production of the living wasps.

### 2.4. Data Analysis

The measurements were evaluated for the different investigated plants (lovage and fennel) and the two wasp species. The measured and evaluated parameters (body temperature, temperature excess, endothermic heat production) were analyzed in dependence on temperature and solar radiation. The calculations were done with MS Excel (Microsoft Corporation, Redmond, WA, USA) and with Origin 2018 software (OriginLab, OriginLab Corporation, Northampton, MA, USA). Curve fitting and statistics were done with Origin and Statgraphics (Statgraphics Centurion XVI, StatPoint Technology Inc., The Plains, VA, USA) software. First, “general linear model (GLM)” statistics were performed to test the influence of the ambient temperature and solar radiation on the measured and calculated parameters. Furthermore, simple linear regressions in combination with an ANOVA were performed to represent and test the dependence of the parameters on ambient temperature and to compare the species. The average values for the evaluated parameters mentioned in the results derive from the fit curves. All statistical details are presented in the supplementary information (Tables S1–S3). To determine the main parameter which influences the wasps’ body temperature most, we calculated a simple model containing radiation and ambient temperature and tested the influence with an ANOVA. Additionally, we compared the data of the *Polistes dominula* foragers on fennel in Austria with data of water-collecting *Polistes dominula* from the study of Kovac et al. [1]. Wasps in that study had been measured at the same location and in the same season (August).

## 3. Results

We started the measurements early in the morning when the first wasps appeared at the flowers and measured until the evening. The wasps did not forage at an ambient temperature below 20 °C. For a foraging bout, they remained at the flower for several minutes, where they were crawling on the inflorescence and made short flights between the flowers.

### 3.1. Operative Temperature

**Figure 2:**
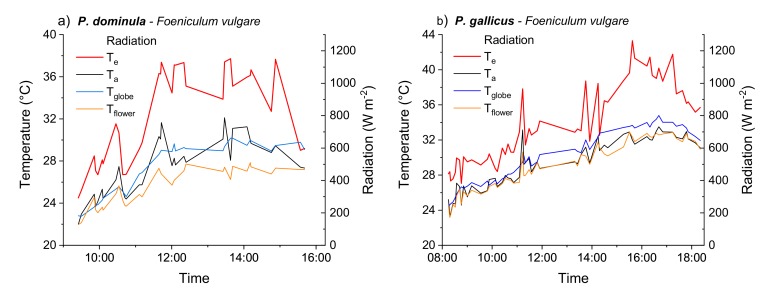
Time course of the operative temperature (T_e_) measured with a dead wasp attached to an inflorescence, of microclimate data (T_a_ = ambient temperature 1 cm beside the wasp, T_flower_ = temperature in the inflorescence, radiation = solar radiation in the inflorescence), and of macroclimate data (T_globe_ = temperature in shade beside the measurement location in 2 m height). (**a**) *Polistes dominula* in Gschwendt/Austria, and (**b**) *Polistes gallicus* in Sesto Fiorentino/Italy, measured at fennel.

The operative temperature (T_e_, thorax temperature of a dead wasp) and the corresponding microclimate conditions during a typical measuring day are shown in Figure 2 for each of the two species. The T_e_ took a similar course as the other parameters (temperature and radiation) but was always elevated above the other measured temperatures. The T_e_ correlated best with the ambient temperature (T_a_) measured in the immediate vicinity of the dead wasps, as shown in Table S1.

### 3.2. Body Temperature

**Figure 3:**
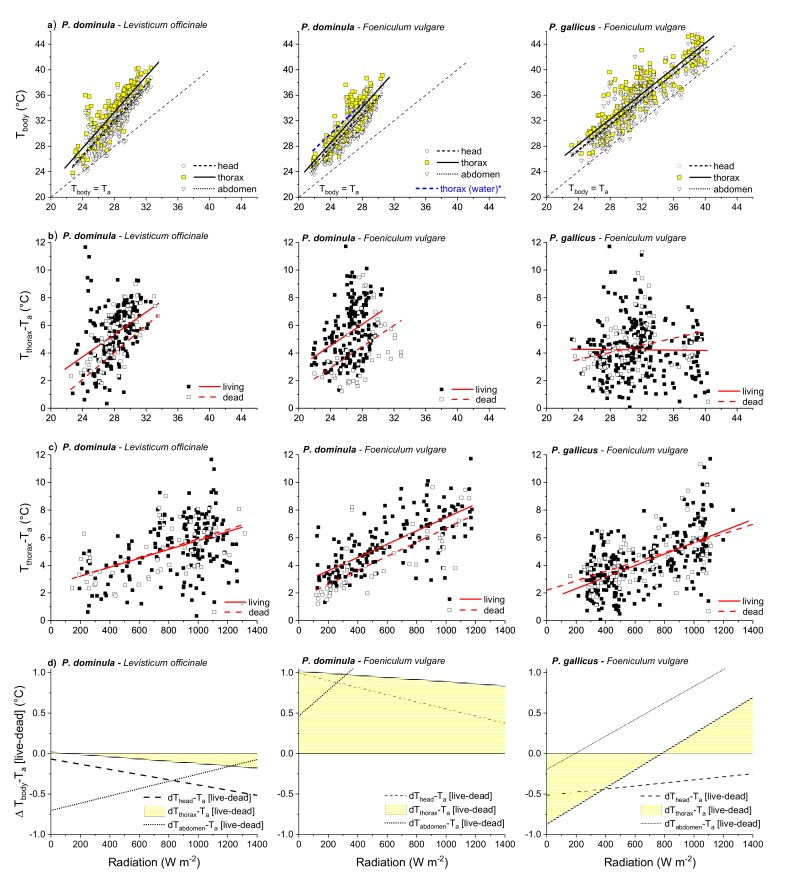
Body temperature (**a**), thorax temperature excess (**b** and **c**), and degree of endothermy (**d**) in dependence on environmental parameters, of foraging *Polistes dominula* and *Polistes gallicus* on lovage and fennel. T_a_ = ambient temperature 1 cm beside the wasps.

The temperature of the three body parts was always higher than that of the ambient air (T_a_) and increased with it in both species, as shown in Figure 3a (for fit curves and statistical details see Tables S2 and S3). The temperature of the thorax was in the range of about 24 to 45 °C, as shown in Figure 3a. For example, the temperature of the *Polistes dominula* thorax increased from 25.7 °C (T_a_ = 22 °C) to 36.9 °C (T_a_ = 30 °C) when foraging on fennel. In *Polistes gallicus* the temperature of the thorax increased from 26.3 °C (T_a_ = 22 °C) to 34.2 °C (T_a_ = 30 °C) and 44.2 °C (T_a_ = 40 °C). In both species, the temperature of the head and of the abdomen was always somewhat lower than that of the thorax but nevertheless was mostly elevated above the ambient air, as shown in Figure 3a.

The statistical analysis with a general linear model, as shown in Table S3, revealed that the thoracic temperature in *Polistes gallicus* depended more strongly on environmental conditions than in *Polistes dominula*. The slope of increase (in thorax temperature) with increasing T_a_ differed between flowers and species. For *Polistes dominula* and *Polistes gallicus* foraging on the same flower (fennel) we calculated a significant difference of both the intercept and slope (*p* < 0.0001, ANOVA). The regression lines of *Polistes dominula* foraging on lovage and fennel differed only in the intercept (*p* < 0.001, ANOVA) but not in the slope of the thorax temperature.

### 3.3. Thorax Temperature Excess

The difference between the thoracic temperature and the ambient temperature (temperature excess T_th_-T_a_) was in the range of about 0 to 12 °C, as shown in Figure 3b. In foraging *Polistes dominula* on lovage and fennel it increased strongly, in both living and dead wasps, with ambient temperature (*p* < 0.0001, ANOVA). The living wasps’ temperature excess was clearly above that of the dead ones. However, in *Polistes gallicus* it increased only in dead (*p* < 0.01, ANOVA) but not in living wasps (*p* > 0.05, ANOVA) with ambient temperature.

The thoracic temperature excess increased in all experiments with increasing solar radiation, as shown in Figure 3c (*p* < 0.0001, ANOVA). However, there was nearly no difference between living and dead wasps in *Polistes dominula* on lovage and in *Polistes gallicus* on fennel but a small and constant difference in *Polistes dominula* foraging on fennel. A model calculation, as shown in Figure S3, and an ANOVA, as shown in Table S3, revealed that the effect of the radiation on the thoracic temperature excess was stronger than that of the ambient temperature.

The difference between the living and the dead wasps’ temperature excess is a measure for their endothermic effort. We therefore calculated the difference of the living and the dead wasps’ body temperature excess (means of head, thorax, and abdomen; Δ T_body_-T_a_ [live-dead]) (1). Figure 3d visualizes the part of temperature increase above T_a_ resulting from endogenously-generated heat in dependence on solar radiation. The results revealed a quite different endothermic behavior. In general, endothermy was small. In *Polistes dominula* foraging on lovage, no endothermic heat production could be detected. However, when foraging on fennel they exhibited a nearly constant endothermic activity of about 1 °C. In *Polistes gallicus* foraging on fennel, weak endothermy was only observed at a radiation intensity higher than 800 W m^−2^.

## 4. Discussion

We investigated the thermoregulatory behavior of two Polistine wasps (*Polistes dominula* and *Polistes gallicus*) in different climate regions, in order to reveal adaptations to their specific environmental conditions. We wanted to show exemplarily the relation between microclimate parameters and body temperature regulation. Although it is known that Polistine wasps are capable of endothermic heat generation [3,4,5], their thermoregulatory behavior during foraging under natural conditions is hardly explored. Kovac et al. [1] investigated the thermoregulation of water foraging paper wasps (*Polistes dominula*) and presented some data on *Polistes dominula* foraging nectar on raspberry and plant sap on rhubarb [1,20]. However, there exist no comprehensive studies on the thermal behavior of nectar foraging paper wasps. To our knowledge, such data are completely missing in *Polistes gallicus*. Our study showed that both species avoid foraging at temperatures below 20 °C. However, when they were foraging the thermoregulatory behavior differed somewhat between species, and also within species during foraging on the different flowers, as shown in Figure 3. The highest thoracic temperature was observed in *Polistes gallicus* foraging on fennel, as shown in Figure 3a. It is remarkable that these wasps foraging at a really high ambient temperature of 40 °C still had an elevated thoracic temperature, reaching 44 °C on average. This is quite close to their critical thermal maximum of 47.6 °C [1]. One might suggest that, similar to Vespine wasps [1,21,22], a high body temperature improves their agility and enables faster foraging. However, for proper take off and flight a thoracic temperature higher than about 30 °C is necessary in wasps and bees e.g., [6,7,8,9]; a thoracic temperature above 40 °C would probably not significantly improve the foraging and flight capability. While the achievable lift (power output) increases with flight muscle temperature up to about 38–39 °C, it decreases at higher muscle temperatures [23,24].

The comparison of the two species foraging on the same flower, that is fennel (but in different habitats), revealed significant differences in the mean thoracic temperature, as shown in Figure 3a, (*p* < 0.0001, ANOVA). The higher temperature of *Polistes dominula* might be explainable by their higher weight (*P. dominula*: 80 ± 15 mg, *P. gallicus*: 44 ± 11 mg; [10]). In Vespine wasps the thermoregulation depends on body mass, but not in a simple linear relation [2]. In species with a weight between 50 to 100 mg the endothermic capability increases strongly, but above 100 mg there is only a small increase of the wasps’ thorax temperatures with their weight. However, Vespine wasps exhibit a much higher endothermic performance than Polistine wasps [1] and it is doubtful whether this dependence of endothermic performance on body mass could be applied in Polistine wasps.

A comparison of *Polistes dominula* foraging on fennel with water foraging *Polistes dominula* in the study by Kovac et al. [1] revealed that the mean thoracic temperature of the water foraging wasps was significantly higher (*p* < 0.001, ANOVA). These investigations were conducted in the same season (August) and at the same location, which facilitates comparison. A possible explanation for the difference in body temperature could be the different duration of the foraging stays. In both cases they had an elevated thoracic temperature after the flight. The water collecting wasps needed only a short time to drink the water (about 16 s). They did not cool down much because of their short foraging duration. Additionally, they probably wanted to keep the thorax temperature high enough (i.e., the flight muscles activated) to be ready for a quick take-off. The nectar foragers, by contrast, remained at the inflorescence for several minutes. They sucked up the nectar of one flower and crawled to the next one, and so had to spend more time on foraging.

The thermoregulatory behavior differed somewhat between species and also within species during foraging on the different plants, as shown in Figure 3. Both species’ body temperature depended strongly on the ambient temperature, but the slope of the temperature increase was steeper in *Polistes dominula* on lovage and fennel (for the thorax), so that one might expect a higher endothermic performance. However, a further analysis of the data could not confirm this. In these measurements the difference between thorax temperature and T_a_ (temperature excess T_th_-T_a_) increased at higher T_a_, as shown in Figure 3b,c, in *Polistes dominula*. In actively thermoregulating insects one would rather expect the opposite, a higher excess at lower T_a_ [25]. In foraging small ecto- and endothermic insects, solar radiation has a great influence on their body temperature, e.g., [1,21,22]. Figure 3b,c show that the thoracic temperature excess increased in dependence on both T_a_ and solar radiation. A model calculation including both environmental parameters, i.e., T_th_-T_a_ = A + B1 × T_a_ + B2 × Radiation, revealed that radiation contributed the main part to this temperature increase, as shown in Figure S3 and Table S3. The effect of T_a_ on the temperature excess (T_th_-T_a_) seems to be an indirect one, because we found an increase of T_a_ with radiation, as shown in Figure S1 (*p* < 0.0001). It has to be expected that sunshine not only increases the insects’ body temperature but also the air temperature over time.

With our dead wasps operating as an “operative temperature model” (e.g., [1,15,16,17,18]), we were able to differentiate between the heat gain from solar radiation and the endothermically-generated heat. Figure 3c and Figure S2 show that the thoracic temperature excess depends strongly on solar radiation. In a further analysis we calculated the difference between the living and the dead wasps’ temperature excess, as shown in Figure 3d. This way we got the wasps’ real average endothermic performance. The results revealed that only in two cases there was a small endothermic action. However, it was different between the species and foraging conditions, as shown in Figure 3d. The endothermic action of *Polistes gallicus* was weak and somewhat differentiated. When foraging on fennel, they changed from an ectothermic state to a weakly endothermic state at higher solar radiation, as shown in Figure 3d. This is a surprising finding because at high radiation levels, and therefore also high ambient temperatures (which were measured during these experiments, as shown in Figure S1), an additional heat generation seems not necessary, as the wasps’ thorax temperature was above 40 °C, as shown in Figure 3a. Foraging *Polistes dominula* on lovage showed no endothermic heat production, but when the same species was foraging on fennel, a small but constant endothermic performance was observed, though the inflorescences of these two Apiales are quite similar and the wasps exhibited a similar foraging behavior. After landing, they looked for nectar and crawled from one flower to the next one or made short flights and left the flower after some minutes. However, one has to keep in mind that the plants might have been in slightly different microhabitats and the specifics of the location, not the plant species, could have been related to the endothermy. Additionally, the attachment of the dead wasp for measuring the operative temperature has also an effect on the measured temperature. Small deviations in the adjustment at the inflorescence could be responsible for unexpected differences in the calculated endothermic performance. These considerations point to the fact that the accuracy of the T_e_ model is limited. Nevertheless, it allows the clear statement that the thermoregulatory behavior of the Polistine wasps is quite different from that of Vespine wasps, which show pronounced endothermy during foraging [1,2,8,11,13,21,22]. At present there are no indications that *Polistes gallicus* and *Polistes dominula* change the degree of endothermy in dependence on differing nectar reward, as is observed by sucrose foraging Vespine wasps [11,21,22].

## 5. Conclusions

Polistine wasps (*Polistes dominula* and *Polistes gallicus*) are capable of endothermic heat production, but their endothermic performance during foraging on nectar is very low. To achieve optimal foraging temperatures, they preferably use solar radiation to increase their body temperature. They avoid a high energetic effort and this way reduce their foraging costs.

## Supplementary Materials

The following are available online at https://www.mdpi.com/2075-4450/10/7/187/s1. Figure S1: Temperature and radiation conditions during foraging of *Polistes dominula* and *Polistes gallicus* on lovage and fennel. T_a_ increased with radiation intensity. Figure S2: Thorax temperature excess of foraging *Polistes dominula* and *Polistes gallicus* on lovage and fennel according to different radiation categories. Figure S3: Thorax temperature excess of living (a) and dead (b) *Polistes dominula* and *Polistes gallicus* on lovage and fennel. A model calculation including both environmental parameters, i.e., T_th_-T_a_ = A + B1*T_a_ + B2*Radiation, revealed that radiation contributed the main part to this temperature increase (Figure S3; Table S3). Black symbols represent measured values and red symbols represent the model results for radiation-corrected values. For statistical details see Supplementary Table S3. Table S1: Statistical details of the correlation between the microclimate data (T_a_, ambient temperature; T_flower_, temperature in inflorescence; T_globe_, temperature weather station and radiation) and the operative temperature model (T_e_), given is R^2^/P/N. Table S2: Statistical details and the fit parameters of linear regressions of all body parts and species. Table S3: Summary of multifactorial ANOVA statistics for the thorax temperature excess (T_th_-T_a_) in Figure 3b.
