# Supplementary material for: The Thermoregulatory Behavior of Nectar Foraging Polistine Wasps (Polistes dominula and Polistes gallicus) in Different Climate Conditions
Source: Insects. 2019 Jun 27;10(7):187. doi: 10.3390/insects10070187 (PMC6681210; doi:10.3390/insects10070187)

insects

MDPI

Article

The Thermoregulatory Behavior of Nectar Foraging Polistine Wasps (*Polistes dominula* and *Polistes gallicus*) in Different Climate Conditions

Helmut Kovac *, Helmut Käfer and Anton Stabentheiner *

[1] Institute of Biology, University of Graz, 8010 Graz, Austria;

* Correspondences: helmut.kovac@uni-graz.at (H.K.); anton.stabentheiner@uni-graz.at (A.S.)

Received: 23 May 2019; Accepted: 22 June 2019; Published: date

Supplementary Materials

Table 1. Statistical details of the correlation between the microclimate data (T_a, ambient temperature; T_{flower}, temperature in inflorescence; T_{globe}, temperature weather station and radiation) and the operative temperature model (T_e), given is R^2/P/N.

Species	T_a	T_{flower}	T_{globe}	radiation
P. dominula	0.81458/0.0001/116	0.59829/0.0001/116	0.66402/0.0001/52	0.42698/0.0001/116
P. gallicus	0.84832/0.0001/85	0.83174/0.0001/85	0.84631/0.0001/85	0.16862/0.0001/85

insects

Table S2. Statistical details and the fit parameters of linear regressions of all body parts and species.

Figure	Species	Flower/body part	Parameter A	Parameter B	R^2	P	N
Figure 3a	*P. dominula*	*lovage*					
		head	-6.52726	1.38051	0.73473	<0.0001	180
		thorax	-5.95509	1.4034	0.70661	<0.0001	180
		abdomen	-5.90242	1.34284	0.74344	<0.0001	180
	P. dominula	*fennel*					
		head	-3.63029	1.31056	0.71592	<0.0001	162
		thorax	-4.80459	1.38879	0.67883	<0.0001	162
		abdomen	-5.08199	1.346	0.73992	<0.0001	162
		water					
		thorax	1.40319	1.18965	0.16862	<0.0001	60
	P. gallicus	*fennel*					
		head	3.578	0.9943	0.81277	<0.0001	263
		thorax	4.38022	0.99513	0.78421	<0.0001	263
		abdomen	3.09637	1.00644	0.79859	<0.0001	263
not shown	*P. dominula**	*lovage*					
		head	-12.16368	1.58158	0.88818	<0.0001	64
		thorax	-10.80405	1.56682	0.87858	<0.0001	64
		abdomen	-11.9229	1.56013	0.85083	<0.0001	64
	*P. dominula**	*fennel*					
		head	-4.01332	1.27794	0.69992	<0.0001	52
		thorax	-6.33345	1.38548	0.75847	<0.0001	52
		abdomen	-1.46829	1.14549	0.76475	<0.0001	52
	*P. gallicus**	*fennel*					
		head	-1.81774	1.18405	0.87496	<0.0001	85
		thorax	0.21001	1.13863	0.84832	<0.0001	85
		abdomen	-0.71786	1.11614	0.87828	<0.0001	85
Figure 3b	*P. dominula*	*lovage*					
		$T_{thorax}-T_a$	-5.95509	0.4034	0.16239	<0.0001	180
		$T_{thorax}-T_a$*	-9.64395	0.48861	0.4582	<0.0001	64
	P. dominula	*fennel*					
		$T_{thorax}-T_a$	-4.80459	0.38879	0.13787	<0.0001	162
		$T_{thorax}-T_a$*	-6.33345	0.38548	0.18367	<0.0001	52
	P. gallicus	*fennel*					
		$T_{thorax}-T_a$	4.38022	-0.00487	-0.00374	>0.05	263
		$T_{thorax}-T_a$*	0.41848	0.12913	0.06941	<0.01	85
Figure 3c	*P. dominula*	*lovage*					
		$T_{thorax}-T_a$	2.57321	0.00322	0.17135	<0.0001	180
		$T_{thorax}-T_a$*	2.55625	0.00336	0.32607	<0.0001	64
	P. dominula	*fennel*					
		$T_{thorax}-T_a$	2.64198	0.00482	0.53159	<0.0001	162
		$T_{thorax}-T_a$*	1.6017	0.00508	0.55684	<0.0001	52
	P. gallicus	*fennel*					
		$T_{thorax}-T_a$	1.47665	0.00418	0.35314	<0.0001	263
		$T_{thorax}-T_a$*	2.19173	0.00341	0.25382	<0.0001	85

* dead wasps

Table S3. Summary of multifactorial ANOVA statistics for the thorax temperature excess (T_{th}-T_a) in Figs 3b.

**P. dominula - lovage**	**Sum of squares**	**DF**	**Mean square**	**F-Quotient**	**P-Value**
Covariable					
Radiation	12.3507	1	12.3507	4.23	<0.05
Main effect					
Temperature	341.235	78	4.37481	1.5	<0.05
Residuals	291.739	100	2.91739		
Total (corrected)	767.681	179			
**P. dominula - fennel**					
Covariable					
Radiation	127.432	1	127.432	67.67	<0.0001
Main effect					
Temperature	126.645	64	1.97883	1.05	>0.05
Residuals	180.779	96	1.88312		
Total (corrected)	661.129	161			
**P. gallicus - fennel**					
Covariable					
Radiation	170.38	1	170.38	70.63	<0.0001
Main effect					
Temperature	328.193	114	2.87889	1.19	>0.05
Residuals	354.619	147	2.41237		
Total (corrected)	1057.96	262			

Figure S1. Temperature and radiation conditions during foraging of *Polistes dominula* and *Polistes gallicus* on lovage and fennel. T_a increased with radiation intensity.

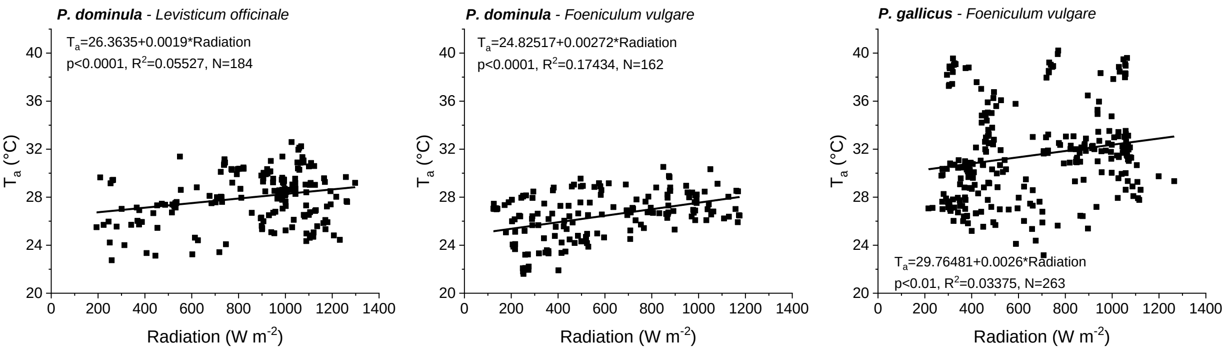

Figure S2. Thorax temperature excess of foraging *Polistes dominula* and *Polistes gallicus* on lovage and fennel for the different radiation categories.

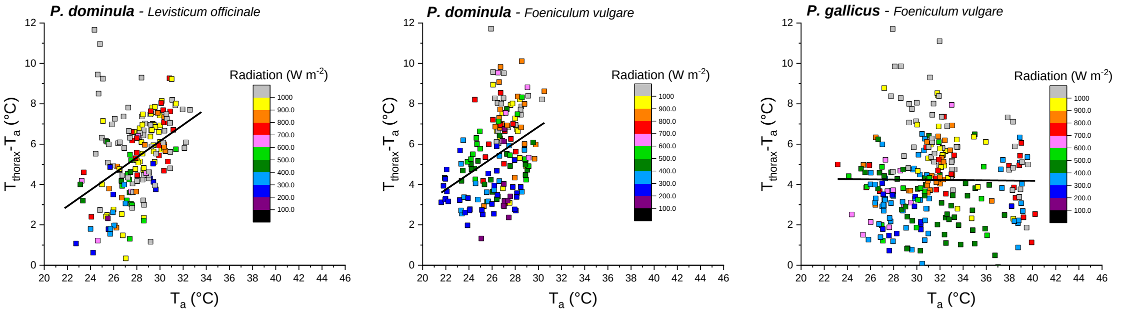

insects

Figure S3. Thorax temperature excess of living (**a**) and dead (**b**) *Polistes dominula* and *Polistes gallicus* on lovage and fennel. A model calculation including both environmental parameters, i.e. T_{th}-T_a = A + B1*T_a + B2*Radiation, revealed that radiation contributed the main part to this temperature increase (Figure S3; Table S3). Black symbols represent measured values and red symbols represent the model results for radiation-corrected values. For statistical details see Supplementary Table S3.

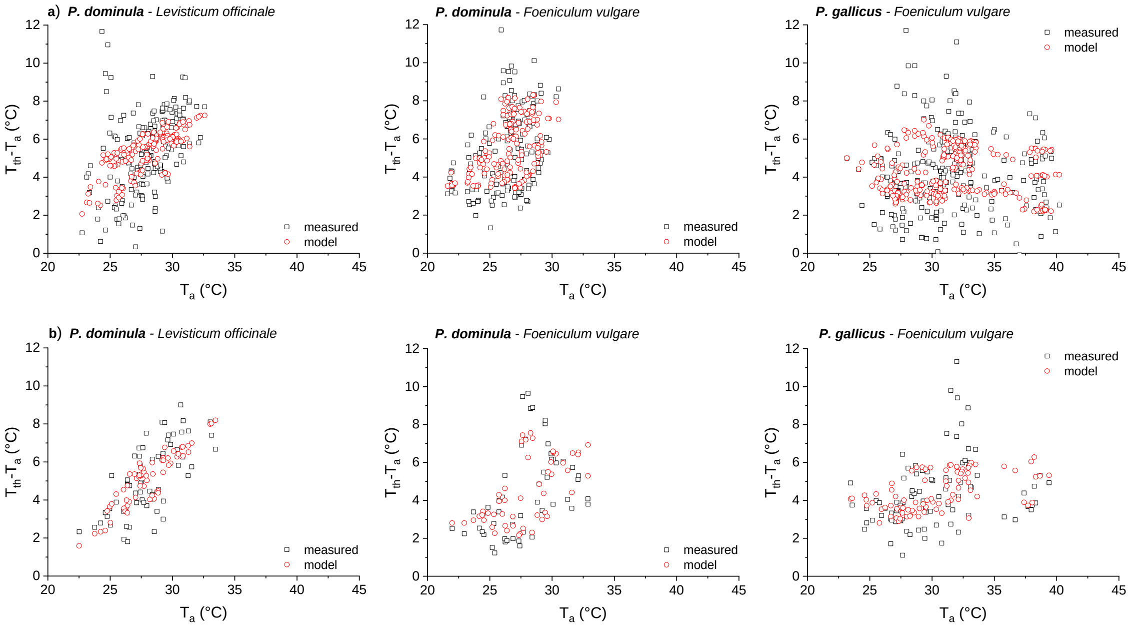

Supplement: Supplementary file 1 [file insects-10-00187-s001.pdf]
